# The Spread of Consumption

**Published:** 1890-08

**Authors:** 


### THE SPREAD OF CONSUMPTION.

Dr. G. M. Brown, in the *Medical Bulletin* for May, makes an earnest appeal to all who are in authority, or who have any influence over patients suffering with pulmonary phthisis, to prevent the spread of the disease. No healthy person, especially no healthy young person, should be permitted to occupy the same room at night with a patient afflicted with bacillary phthisis. Physicians in attendance upon such cases should warn the members of the household of the dangers of too close contact with the sick. If the one already attacked cannot be saved, it is possible, at least, to prevent others from being sacrificed. The germ may remain latent for years after infection, but it is present, ready to break out at the first favorable opportunity. If every physician would do his whole duty in this matter, consumption would be less common than it is now.

